# Using Fear and Anxiety Related to COVID-19 to Predict Cyberchondria: Cross-sectional Survey Study

**DOI:** 10.2196/26285

**Published:** 2021-06-09

**Authors:** Xue Wu, Nabi Nazari, Mark D Griffiths

**Affiliations:** 1 School of Economics and Management Kaili University Guizhou China; 2 Department of Psychology Faculty of Literature and Human Sciences Lorestan University Khorramabad Iran; 3 International Gaming Research Unit Psychology Division Nottingham Trent University Nottingham United Kingdom

**Keywords:** COVID-19, cyberchondria, COVID-19 fear, COVID-19 anxiety, anxiety sensitivity, anxiety, intolerance of uncertainty, mental health, survey, SEM

## Abstract

**Background:**

Studies have highlighted that fear and anxiety generated by COVID-19 are important psychological factors that affect all populations. There currently remains a lack of research on specific amplification factors regarding fear and anxiety in the context of the COVID-19 pandemic. Despite established associations between anxiety sensitivity, intolerance of uncertainty, and cyberchondria, empirical data investigating the associations between these three variables, particularly in the context of the COVID-19 pandemic, are currently lacking. Urgent research is needed to better understand the role of repeated media consumption concerning COVID-19 in amplifying fear and anxiety related to COVID-19.

**Objective:**

This study investigated the associations between fear of COVID-19, COVID-19 anxiety, and cyberchondria.

**Methods:**

Convenience sampling was used to recruit respondents to participate in an online survey. The survey, which was distributed via social media and academic forums, comprised the Cyberchondria Severity Scale, Fear of COVID-19 Scale, Coronavirus Anxiety Scale, Anxiety Sensitivity Index, and Intolerance of Uncertainty Scale. Multiple mediation analyses were conducted using structural equation modeling.

**Results:**

A total of 694 respondents (males: n=343, females: n=351) completed the online survey. The results showed that fear and anxiety generated by COVID-19 predicted cyberchondria (fear: β=.39, SE 0.04, *P*<.001, *t*=11.16, 95% CI 0.31-0.45; anxiety: β=.25, SE 0.03, *P*<.001, *t*=7.67, 95% CI 0.19-0.32). In addition, intolerance of uncertainty and anxiety sensitivity mediated the relationship between fear and anxiety generated by COVID-19 with cyberchondria. In a reciprocal model, the standardized total effects of cyberchondria on fear of COVID-19 (β=.45, SE 0.04, *P*<.001, *t*=15.31, 95% CI 0.39-0.51) and COVID-19 anxiety (β=.36, SE 0.03, *P*<.001, *t*=11.29, 95% CI 0.30-0.41) were statistically significant, with moderate effect sizes. Compared to males, females obtained significantly higher scores for cyberchondria (*t*_1,692_=–2.85, *P*=.004, Cohen *d*=0.22), COVID-19 anxiety (*t*_1,692_=–3.32, *P*<.001, Cohen *d*=0.26), and anxiety sensitivity (*t*_1,692_=–3.69, *P*<.001, Cohen *d*=0.29).

**Conclusions:**

The findings provide a better understanding of the role of COVID-19 in amplifying cyberchondria. Based on these results, cyberchondria must be viewed as a significant public health issue. Importantly, increasing awareness about cyberchondria and online behavior at both the individual and collective levels must be prioritized to enhance preparedness and to reduce the adverse effects of current and future medical crises.

## Introduction

### Background

The COVID-19 outbreak is more of a global emergency than a medical challenge. Research highlights the intense and broad spectrum of psychosocial ramifications that pandemics can inflict on the general population [1]. Fear of COVID-19 and COVID-19 anxiety, coupled with quarantine and isolation [2], can generate specific negative psychological responses such as maladaptive behaviors, emotional distress, and avoidance reactions among both general and patient populations [3,4]. Compared with previous pandemics (eg, severe acute respiratory syndrome [SARS]), psychological distress and anxiety disorders related to the increasingly widespread use of the internet are relatively novel problems in psychiatric and medical settings. The internet can be a useful source of health information [5] and has become increasingly prevalent among all members of the public. However, repeated media exposure to pandemic-related information and excessive searching for health-related information on the internet can significantly exacerbate anxiety and create an escalating pattern of psychological distress that is difficult to break. This has been termed “cyberchondria” [6,7].

### Cyberchondria in the Context of the COVID-19 Pandemic

Cyberchondria has been defined as “anxiety resulting from a health-related search online” [8,9]. Cyberchondria is conceptualized as a multidimensional construct, including repetitive (excessive) online searching for health-related information, distress (increased negative affect), compulsion (interruption of daily routine), and reassurance seeking. Seeking health-related information on the internet to reduce anxiety may result in more anxiety or distress [10]. In the latest (fifth) edition of the Diagnostic and Statistical Manual of Mental Disorders (DSM-5), individuals can receive one of two anxiety-related diagnoses. Somatic symptom disorder refers to anxiety concerning health diagnoses in the context of significant somatic symptoms whereas illness anxiety disorder refers to a health anxiety diagnosis without somatic symptoms. Individuals affected by these mental health problems tend to misinterpret minor bodily sensations or symptoms as if they were severe illnesses [11]. When individuals with these diagnoses use online networks to search for health-related information, they are classified as having cyberchondria [12]. As a safety behavior, cyberchondria (ie, anxiety when searching online for health-related information) may fuel psychopathological vulnerabilities [12]. This indicates a strong relationship between health anxiety and cyberchondria [13,14]. Individuals with elevated health anxiety exhibit higher anxiety levels during and after online health-related searches and report more frequent and longer online searches than those with lower levels of health anxiety [15,16]. These discrepancies between the purpose and outcome of cyberchondria may complicate our understanding of patterns of cyberchondria. In addition to anxiety, fear is a substantial motivating factor in seeking health-related information [17]. Cyberchondria may also exacerbate fear of illness and have a negative impact on relationships with primary care physicians. Cyberchondria is also associated with accessing increased health resources, as measured by the number of visits to general practitioners and other health professionals [18].

### Fear in the Context of the COVID-19 Pandemic

Fear, generated by trauma-related stimuli, is a prominent emotion in psychopathology [19]. Fear can be viewed as an adaptive response to threat, which can be a motivating factor that facilitates protective and preventive behavior among individuals to avoid infection and follow pandemic-related health instructions. However, for individuals who experience fear intensely, it may result in an elevated risk perception. Consequently, this adaptive response to fear becomes maladaptive when these emotional responses fail to provide accurate information. Prior experience, cognitive and attentional biases, and mental disorders can all generate faulty appraisals of the physical and social environment, leading to maladaptive emotional reactions. In the context of the current COVID-19 pandemic, fear and anxiety can elicit additional media consumption [20]. In addition, the consumption of pandemic-related media coverage may be an important factor that is associated with anxiety and psychological distress [7,21]. For example, exposure to warning messages, as well as inaccurate and misleading information concerning life-threatening aspects of COVID-19 during online searching, can exacerbate anxiety and worries related to the pandemic [22]. Moreover, a significant positive association has been found between anxiety resulting from online health searches for oneself and anxiety resulting from online health searches for others [8]. Fear of self-infection or infecting family members is one of the most common reactions to pandemics [23,24] and can result in health anxiety, worries, specific phobias, and psychological distress [25-27]. There is also a strong relationship between cyberchondria and health anxiety [28]. Health anxiety can motivate excessive or repeated health-related information seeking on the internet, which can amplify anxiety or distress (eg, fear) [9,15,29]. Therefore, individuals with cyberchondria may be anxious about the health of family members, attempt to diagnose them online, and/or take additional measures as a consequence of their fear of COVID-19.

### Uncertainty and Anxiety Sensitivity During the COVID-19 Pandemic

The current pandemic has caused much uncertainty about many different aspects of daily life. Intolerance of uncertainty is recognized as a strong predictor of cyberchondria. In times of uncertainty, reducing uncertainty has a central role in motivating searching for health information on the internet [30]. In addition, individuals with a higher level of intolerance of uncertainty exhibit prospective anxiety due to dispositional fear of unknown future events [31,32]. Individuals with a higher intolerance of uncertainty levels may perceive uncertain situations as both threatening and aversive. Therefore, individuals engage in uncertainty-reducing behaviors (eg, repeatedly seeking reassurance due to worries) to moderate the perceptions of uncertainty and threat [33].

Anxiety sensitivity, defined as “the fear of sensations of anxious arousal based on beliefs about their harmful consequences,” is conceptualized as a cognitive-emotional individual difference factor of the fear related to bodily sensations [34]. Experimental studies have demonstrated a positive association between anxiety sensitivity and oversearching of medical information. Compared to individuals with generalized anxiety disorder, anxiety sensitivity is recognized as a potential risk factor for increased anxiety related to COVID-19 [35]. Research also indicates that accurate knowledge about pandemics may be associated with anxiety. During the COVID-19 pandemic, it is possible that such individuals search for information and medical news related to COVID-19 with high sensitivity to anxiety, which leads to an increase in their fear.

### Purpose of This Study

Understanding COVID-19 pandemic–related psychopathology development is limited due to numerous individual and contextual factors. There is currently a lack of research on specific amplification factors regarding fear and anxiety in the context of the COVID-19 pandemic. Cyberchondria is a maladaptive behavioral pattern, more likely during public health crises such as the COVID-19 pandemic. Research is urgently needed to better understand the role of repeated media consumption concerning COVID-19 [36]. Despite established associations between anxiety sensitivity, intolerance of uncertainty, and online medical information seeking [37-39], there is currently a lack of empirical data concerning the associations between anxiety sensitivity, intolerance of uncertainty, and cyberchondria, particularly in the context of the COVID-19 pandemic.

Therefore, this study investigated the associations between fear of COVID-19, COVID-19 anxiety, and cyberchondria. Using structural equation modeling (SEM), a mediation analysis was carried out to investigate the underlying mechanism between cyberchondria and fear of COVID-19, COVID-19 anxiety, intolerance of uncertainty, and anxiety sensitivity. In addition, an evaluation of the reverse mediation model of the association between the study variables was also carried out. The study may potentially contribute to a better understanding of the pandemic in relation to cyberchondria. Moreover, the findings provide additional insight into cyberchondria and the pandemic, providing important information to clinical practitioners and policymakers. It was hypothesized that intolerance of uncertainty and anxiety sensitivity would mediate the association between fear of COVID-19 and COVID-19 anxiety on cyberchondria.

## Methods

### Inclusion Criteria

The eligibility criteria included (1) age >18 years, (2) not hospitalized or quarantined in the current or a past viral pandemic due to infection, (3) not having (or suspect as having) COVID-19, (4) being able to read and complete an online survey and provide informed consent, (5) fluency in the Persian language, and (6) currently living in Iran. Only completed questionnaires were analyzed.

### Measures

The survey comprised the Cyberchondria Severity Scale, Fear of COVID-19 Scale, Coronavirus Anxiety Scale, Anxiety Sensitivity Index, and Intolerance of Uncertainty Scale.

#### Cyberchondria Severity Scale–Short Form

The Cyberchondria Severity Scale–Short Form (CSS-12) [40] is a 12-item self-report scale designed to assess anxiety attributable to health-related online searches. The items (eg, “If I notice an unexplained bodily sensation, I will search for it on the Internet”) are rated on 5-point scale from 1 (never) to 5 (always). The scale comprises three subscales: compulsion, distress, and mistrust of medical professionals. Higher scores indicate greater cyberchondria. The internal consistency of the CSS-12 in this study was excellent (Cronbach α=.90).

#### Fear of COVID-19 Scale

The Fear of COVID-19 Scale (FCV-19S) [41] is a 7-item unidimensional scale that assesses fear of COVID-19. The items (eg, “I am afraid of losing my life because of COVID-19”) are rated on a 5-point scale ranging from 1 (strongly disagree) to 5 (strongly agree). Higher scores indicate a greater fear of COVID-19. The internal consistency of the FCV-19S in this study was very good (Cronbach α=.83).

#### Coronavirus Anxiety Scale

The 5-item Coronavirus Anxiety Scale (CAS) [42] assesses dysfunctional anxiety associated with COVID-19. The items (eg, “I had trouble falling or staying asleep because I was thinking about the coronavirus”) are rated on a 5-point scale, ranging from 1 (strongly disagree) to 5 (strongly agree). Higher scores are associated with a COVID-19 anxiety diagnosis, impairment, maladaptive coping, and suicidal ideation. The internal consistency of the CAS in this study was excellent (Cronbach α=.90).

#### The Anxiety Sensitivity Index-3

The Anxiety Sensitivity Index-3 (ASI-3) [43] is an 18-item self-report scale that assesses anxiety-related symptoms. Items (eg, “It scares me when my heart beats rapidly”) are rated on a 5-point scale from 0 (not at all) to 4 (very much). Higher scores indicate a more severe anxiety sensitivity level. The internal consistency of the ASI-3 in this study was very good (Cronbach α=.85).

#### Intolerance of Uncertainty Scale-12

The Intolerance of Uncertainty Scale-12 (IUS-12) [44] is a 12-item scale that assesses individuals’ responses to uncertainty. The items (eg, “It frustrates me not having all the information I need”) are rated on a 5-point scale from 1 (not at all characteristic of me) to 5 (entirely characteristic of me). Higher scores indicate greater uncertainty. The internal consistency of the IUS-12 in this study was very good (Cronbach α=.82).

### Participant Recruitment

The study was conducted during the COVID-19 pandemic (October and November 2020) via convenience sampling; hence, all data were collected online because face-to-face data collection was not possible. The participants were recruited over a 6-week period using an online platform to complete the survey. The recruitment process included advertising the study via social media platforms (Instagram, WhatsApp) with links to the survey. In addition, the link was distributed on several academic forums. Once the link was clicked, it led to an informed consent page to be read and agreed upon before proceeding to the survey. Only those who provided informed consent were able to access the survey. The informed consent page included information about the study goals, such as the study’s objectives and confidentiality.

### Sample Size

A priori power analysis for multiple linear regression was calculated using G*Power (Heinrich-Heine-Universität Düsseldorf) to determine the sample size, with an alpha of .025, a power of 0.80, Cohen f^2^ of 0.02, and two predictors [45]. The effect size value (Cohen f^2^=0.02) signifies small effect sizes, according to Cohen’s guidelines [46]. The desired total sample size was 576. In total, 694 participants were recruited in this study, which allowed for a 20% data attrition.

### Ethics

The study, including all assessments and procedures for the study, were reviewed by the National Institute for Medical Research and Development and the Institutional Human Research Ethics Committee. The corresponding author’s institutional review board also approved the research protocol to ensure participant confidentiality, sampling, and informed consent.

### Data Analysis

#### Descriptive Statistics

Descriptive statistics were used to calculate the sample characteristics. Absolute skewness and kurtosis values assessed the normality assumption [47]. Variance inflation factor (VIF) was utilized to examine multicollinearity (1<VIF<3) [48]. Pearson coefficient correlation analysis was carried out to calculate the association between cyberchondria and the study variables. There were no missing values in the assessed variables. Therefore, no imputation method was implemented.

#### Multiple Mediation Analysis

Parallel multiple mediation analysis was conducted using SEM with a 95% CI for indirect effects and 5000 bootstrapping [49]. Once the measurement models were fitted to the data, 2 SEM models were examined. The first SEM investigated the relationships between fear of COVID-19 and COVID-19 anxiety (as independent variables) with cyberchondria. Anxiety sensitivity and intolerance of uncertainty were potential mediators. In the second SEM, the reverse model was examined. An indirect effect was considered statistically significant when the bias-corrected CI does not include zero [50,51]. Cohen f^2^ values of ≥0.15 and ≥0.35 signified approximately moderate to large effect sizes, according to Cohen’s guidelines [46]. SPSS (version 25, IBM Corp) and AMOS (version 24, IBM Corp) were utilized to test hypothesizes (two-tailed), and an alpha level of .05 was used to indicate statistical significance.

## Results

### Descriptive Statistics

Of the 820 returned surveys, 694 met the inclusion criteria and were included in the analysis. Therefore, the sample comprised 694 adults from the general population (males: n=343, 49.4%; females: n=351, 50.6%) , with a mean age of 27.92 years (SD 5.22, range 19-41 years). The demographic characteristics of the sample are shown in Table 1.

There was no significant difference between males and females (χ²=0.09, *P*=.76). The participants were well educated and young. With respect to educational level, 25.8% (n=179) had completed high school, 49.2% (n=341) had a bachelor’s degree, and 25% (n=174) had a master’s and/or higher degree. Compared to males, females had significantly higher scores for cyberchondria (*t*_1,692_=–2.85, *P*=.004, Cohen *d*=0.22), COVID-19 anxiety (*t*_1,692_=–3.32, *P*<.001, Cohen *d*=0.26), and anxiety sensitivity (*t*_1,692_=–3.69, *P*<.001, Cohen *d*=0.29). Compared to females, males had significantly higher scores for intolerance of uncertainty (*t*_1,692_=2.29, *P*=.02, Cohen *d*=0.18). Gender differences for other variables were nonsignificant (*P*>.05) (Table 1).

The univariate normality of the data was checked. Values of skewness and kurtosis were within <|1|, suggesting the absence of severe normality. The VIF values demonstrated no violation of multicollinearity (Table 2). The Pearson coefficient correlation analyses showed a moderate to large correlations between variables (Table 2). Correlation analysis revealed a moderate to large correlation between cyberchondria and fear of COVID-19, COVID-19 anxiety, anxiety sensitivity, and intolerance of uncertainty.

**Table 1:** Demographic characteristics and descriptive statistics of the sample (N=694).

Characteristic		Participants	Statistics^a^	*P* value
**Gender, n (%)**			χ²=0.09	.76
	Male	343 (49.4)		
	Female	351 (50.6)		
**Age group, n (%)**			χ²=11.60	.003
	19-25 years	193 (27.8)		
	26-31 years	266 (38.3)		
	>31 years	235 (33.9)		
Age (years), mean (SD)		27.92 (5.29)	*t*_1,692_=0.82	.41
Cyberchondria, mean (SD)		33.17 (8.04)	*t*_1,692_=–2.85	004
Coronavirus anxiety, mean (SD)		11.91 (2.51)	*t*_1,692_=–3.32	.001
Fear of COVID-19, mean (SD)		15.21 (4.93)	*t*_1,692_=–1.70	.09
Intolerance of uncertainty, mean (SD)		38.82 (9.52)	*t*_1,692_=2.29	.02
Anxiety sensitivity, mean (SD)		28.52 (8.29)	*t*_1,692_= –3.69	<.001

^a^Negative *t* values indicate that females obtained higher scores.

**Table 2:** Correlation matrix of main variables (N=694).

Item	1	2	3	4	5	6	Skewness	Kurtosis	VIF^a^
1. Age	1.00						0.49	–0.99	—^b^
2. Cyberchondria	–0.09^c^	1.00					0.38	0.85	2.51
3. Coronavirus anxiety	0.04	0.36^d^	1.00				0.70	–0.69	1.82
4. Fear of COVID-19	–0.06	0.46^d^	0.29^d^	1.00			0.39	0.75	1.35
5. Intolerance of uncertainty	0.26^d^	0.43^d^	0.31^d^	0.29^d^	1.00		0.65	–0.96	2.23
6. Anxiety sensitivity	­–0.03	0.31^d^	0.27^d^	0.44^d^	0.36^d^	1.00	0.22	–1.02	1.92

^a^VIF: variance inflation factor.

^b^Not applicable.

^c^Correlation significant at the *P*<.05 level (two-tailed).

^d^Correlation significant at the *P*<.01 level (two-tailed).

### Multiple Mediation Analysis

**Figure 1:**
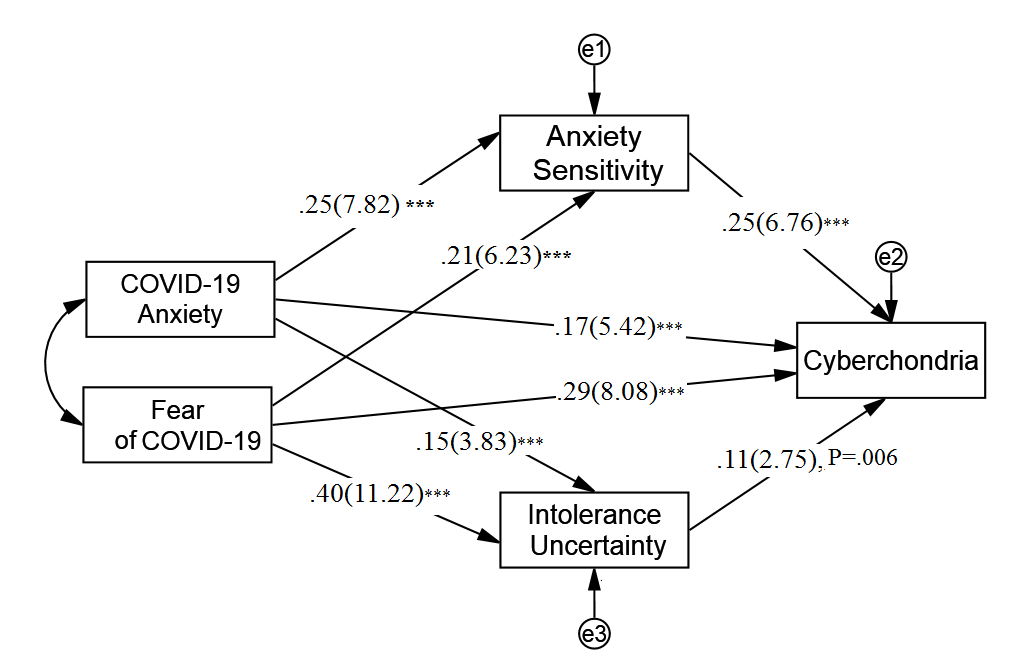
The multiple mediation model. Path coefficient: standardized coefficient (*t* value). ***P<.001 level (two-tailed).

The first SEM mediation analysis showed that fear of COVID-19 and COVID-19 anxiety were significantly associated with cyberchondria via both direct and indirect paths. The standardized total effects for fear of COVID-19 and COVID-19 anxiety on cyberchondria were both statistically significant (fear: β=.39, SE 0.04, *P*<.001, *t*=11.16, 95% CI 0.31-0.45; anxiety: β=.25, SE 0.03, *P*<.001, *t*=7.67, 95% CI 0.19-0.32). Anxiety sensitivity and intolerance of uncertainty were also positively associated with cyberchondria (Figure 1). In addition, for fear of COVID-19 and COVID-19 anxiety, the indirect effects of anxiety sensitivity and intolerance of uncertainty on cyberchondria were significant (Table 3).

**Figure 2:**
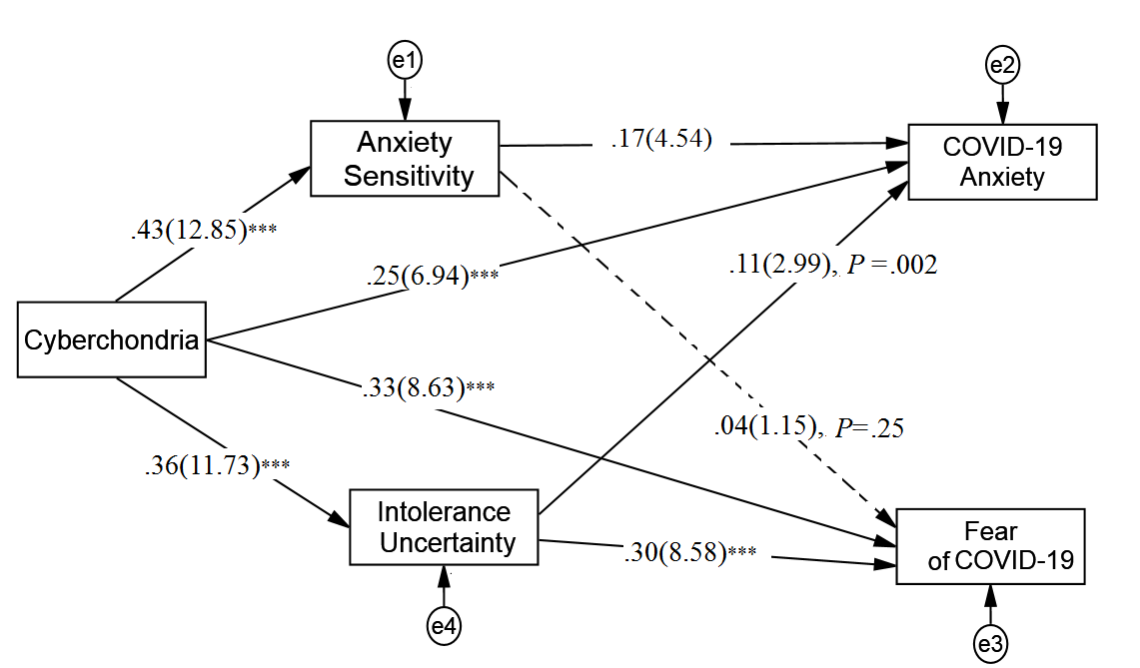
The reciprocal multiple mediation model. Path coefficient: standardized coefficient (*t* value), dash line: nonsignificant path. ***P<.001 level (two-tailed).

In the reciprocal model, the standardized total effects of cyberchondria on both fear of COVID-19 and COVID-19 anxiety were statistically significant (fear: β=.45, SE 0.04, *P*<.001, *t*=15.31, 95% CI 0.39-0.51; anxiety: β=.36, SE 0.03, *P*<.001, *t*=11.29, 95% CI 0.30-0.41. The indirect effects of cyberchondria on COVID-19 anxiety via anxiety sensitivity and intolerance of uncertainty were both significant. However, only cyberchondria’s indirect effect on fear of COVID-19 via anxiety sensitivity was significant (Figure 2). The effect sizes for associations in both mediation models are reported in Table 4.

**Table 3:** Standardized indirect effects of model paths.

Model and indirect path^a^		Beta^b^	SE	*t* value	*P* value	95% CI
**Model 1**						
	Coronavirus anxiety –> intolerance of uncertainty –> cyberchondria	.06	0.02	4.98	<.001	0.04 to 0.09
	Fear of COVID-19 –> intolerance of uncertainty –> cyberchondria	.06	0.01	4.52	<.001	0.03 to 0.08
	Coronavirus anxiety –> anxiety sensitivity –> cyberchondria	.02	0.01	2.23	.03	0.004 to 0.03
	Fear of COVID-19 –> anxiety sensitivity –> cyberchondria	.04	0.02	2.67	.01	0.01 to 0.07
**Model 2 (reciprocal model)**						
	Cyberchondria –> intolerance of uncertainty –> coronavirus anxiety	.07	0.02	4.26	<.001	0.04 to 0.11
	Cyberchondria –> anxiety sensitivity –> coronavirus anxiety	.04	0.01	2.98	.004	0.01 to 0.07
	Cyberchondria –> intolerance of uncertainty –> fear of COVID-19	.02	0.02	1.15	.25	–0.01 to 0.05
	Cyberchondria –> anxiety sensitivity –> fear of COVID-19	.11	0.02	6.99	<.001	0.08 to 0.15

^a^Arrow indicates path direction.

^b^Beta: standardized path coefficient.

**Table 4:** Effect sizes of model paths.

Model and path^a^		Cohen f^2^	*P* value	95% CI
**Model 1**				
	Fear of COVID-19 –> cyberchondria	0.10	<.001	0.06-0.16
	Fear of COVID-19 –> intolerance of uncertainty	0.06	.002	0.03-0.09
	Fear of COVID-19 –> anxiety sensitivity	0.19	<.001	0.13-0.27
	Coronavirus anxiety –> cyberchondria	0.04	.008	0.02-0.07
	Coronavirus anxiety –> intolerance of uncertainty	0.07	.001	0.04-0.11
	Coronavirus anxiety –> anxiety sensitivity	0.03	.04	0.01-0.06
	Intolerance of uncertainty –> cyberchondria	0.08	.002	0.04-0.14
	Anxiety sensitivity –> cyberchondria	0.01	.18	0.001-0.04
**Model 2 (** **reciprocal model** **)**				
	Cyberchondria –> coronavirus anxiety	0.06	.001	0.03-0.10
	Cyberchondria –> fear of COVID-19	0.12	<.001	0.07-0.19
	Cyberchondria –> intolerance of uncertainty	0.23	<.001	0.15-0.31
	Cyberchondria –> anxiety sensitivity	0.16	<.001	0.11-0.25
	Intolerance of uncertainty –> coronavirus anxiety	0.02	.03	0.01-0.05
	Intolerance of uncertainty –> fear of COVID-19	0.002	.65	0.00-0.01
	Anxiety sensitivity –> coronavirus anxiety	0.01	.15	0.001-0.03
	Anxiety sensitivity –> fear of COVID-19	0.11	<.001	0.06-0.17

^a^Arrow indicates path direction.

## Discussion

### Principal Findings

Understanding COVID-19 pandemic–related psychopathological development has been limited due to numerous individual and contextual factors. There is concern that individuals affected by fear and anxiety generated by the pandemic will rapidly outnumber infected cases. This study investigated the impact of the relationship between cyberchondria and the fear and anxiety generated by COVID-19. The study also explored whether the relationships would be mediated by the intolerance of uncertainty and anxiety sensitivity. Results indicated that greater fear and anxiety related to COVID-19 directly predicted cyberchondria [52-54] and indirectly via the mediator variables. Cyberchondria and anxiety generated by COVID-19 was bidirectional. A higher level of cyberchondria directly predicted a higher level of fear and anxiety generated by COVID-19, and indirectly via the mediators.

The mediation analysis empirically showed that intolerance of uncertainty and anxiety sensitivity mediated the associations between fear and anxiety generated by COVID-19 on cyberchondria. In the context of COVID-19, individuals with a higher intolerance of uncertainty levels may search for medical information to reduce uncertainty that results in additional negative experience [55-57]. Intolerance of uncertainty amplifies both threat perception and uncertainty perception [58], which can lead to more engagement in safety behaviors (eg, checking behavior) [59]. Like a vicious circle, seeking health-related information on the internet to reduce uncertainty may be associated with greater levels of uncertainty and therefore amplify health anxiety. These safety-seeking behaviors to reduce uncertainty are often not long lasting. In addition, most uncertainty reduction attempts meet with information that is overly brief and inaccurate, which can lead to greater levels of uncertainty. Moreover, uncertainty-reducing behaviors can lead to greater uncertainty perceptions and/or higher perceived threat severity. During a pandemic, individuals with a high intolerance of uncertainty perceive low-risk situations as highly threatening and report higher anxiety levels [34].

Individuals with high anxiety sensitivity (1) are more afraid of pain and more likely to seek unnecessary treatment for minor pain symptoms [60]; and (2) may misinterpret symptoms, which can result in bodily sensations related to anxiety (eg, sweating, shaky hands) that may be interpreted as severe physical symptoms or illness [61]. Since individuals with elevated anxiety sensitivity interpret anxiety-related bodily sensations to be dangerous, they exhibit increased online medical information-seeking behaviors in an attempt to placate concerns about the origins of anxiety-related bodily sensations. Specifically, this cognitive-affective condition is considered conceptually distinct from anxiety and reflects the fear of anxious arousal symptoms. Anxiety sensitivity is a more robust predictor of posttraumatic symptoms with a moderate to large effect size [62]. On the other hand, the reduction in anxiety sensitivity positively predicts a reduction in the severity of anxiety symptoms [63,64]. This theoretical process is consistent with research that has found the engagement in safety behaviors, including using medical websites to investigate medical symptoms, results in increased levels of health anxiety. With respect to the bidirectional associations between intolerance of uncertainty and anxiety sensitivity, findings suggest that evaluation of both conditions can add to the diagnosis process for individuals with cyberchondria.

This study also found some gender differences. In line with the few studies to date, females reported a higher levels for cyberchondria than males [65]. Females also reported higher levels of COVID-19 anxiety and anxiety sensitivity than males in this study. In line with recent studies, females reported more psychological problems associated with COVID-19 than males [66-68]. Our findings concur with previous studies indicating that females report greater psychological problems and are more likely to develop anxiety symptoms than males [69]. Regarding age, the results suggest that behaviors related to cyberchondria appear to be more prevalent among younger individuals [70,71].

### Limitations

The findings of this study should be interpreted in light of several limitations. This study was conducted during October 2020 at the height of the COVID-19 pandemic in Iran. Therefore, to minimize infection risk, online data collection was utilized rather than a traditional face-to-face method. Online data collection may limit the participation of specific relevant population groups (eg, disadvantaged groups such as those living in poverty who may not have internet access). Therefore, the data do not represent all groups’ views, affecting the generalizability of the study’s findings. However, online data collection tends to provide more honest and truthful responses than offline methods [72]. Moreover, all data were self-reported and are therefore subject to well-established method biases. It should also be noted that the data collected did not include some potentially important variables such as whether (1) the participants were currently working or whether they had lost their job as a result of the pandemic, (2) they and/or their family members had experienced COVID-19, and (3) whether they had financial problems as a result of the pandemic. These are all variables that could be considered in future research when reexamining the variables of this study. Finally, the data were cross-sectional; therefore, determining the true relationships and directions of causality between the study variables is not possible. Future studies would need longitudinal designs to determine true causality.

### Conclusion

Despite these limitations, the findings of the first SEM suggest that anxiety sensitivity and/or intolerance of uncertainty may lead to the development of cyberchondria in the context of the COVID-19 pandemic, with small to moderate effect sizes. In this study, greater fear and anxiety of COVID-19 were associated with greater cyberchondria. However, the reverse SEM demonstrated that cyberchondria is also associated with the study construct, with moderate to large effect sizes. In addition to the pandemic, anxiety sensitivity and intolerance of uncertainty can be critical in increasing or maintaining psychopathological development, as well as physical and psychological dysfunctions [73-75]. Given the co-occurring nature of mental health problems during the current pandemic, disorder-specific interventions may be difficult to justify when the clinical reality is complex and comorbidities are the norm [76]. Many clinical studies have recommended shifting from the traditional specific disorder–focused approach toward a transdiagnostic treatment as an alternative approach. Despite some scholars’ assertions [77], the findings of this study do not justify cyberchondria as a transdiagnostic condition. However, the transdiagnostic treatments or application of the relative modules may be effective in treatment for cyberchondria. For example, higher self-awareness and contextual awareness enable individuals to clearly identify the triggering of negative responses and can help reduce maladaptive cognitive patterns by facilitating awareness or attention toward an object (eg, heartbeat, breathing) in a mindful manner [78,79]. To provide adaptive emotional responding to anxiety-related bodily sensations, self-awareness can be promoted by the mindfulness-based stress reduction therapy or other unified protocols [80].

This study’s findings help to explain how the consequences of the pandemic can be associated with cyberchondria. The role of cyberchondria in the exacerbation of pandemic-related psychological distress can provide further evidence that maladaptive new age issues related to human-internet interaction need further attention from scholars, policymakers, and health care practitioners. Finally, based on the findings here, cyberchondria must be viewed as a significant public health issue. Importantly, increasing awareness about cyberchondria and online behavior at both the individual and collective levels must be prioritized to enhance preparedness and reduce adverse effects associated with the current pandemic and future medical crises.

## Abbreviations

ASI-3: Anxiety Sensitivity Index-3
CAS: Coronavirus Anxiety Scale
CSS-12: Cyberchondria Severity Scale–Short Form
DSM-5: Diagnostic and Statistical Manual of Mental Disorders, fifth edition
FCV-19S: Fear of COVID-19 Scale
IUS-12: Intolerance of Uncertainty Scale-12
SARS: severe acute respiratory syndrome
SEM: structural equation modeling
VIF: variance inflation factor
